# Burden of Antibiotic Resistance at Wolaita Sodo University Comprehensive Specialized Hospital

**DOI:** 10.1155/2022/7272024

**Published:** 2022-06-02

**Authors:** Temesgen Anjulo Ageru, Habtamu Seid, Temesgen Lera Abiso, Abera Kumalo, Temesgen Sidamo, Tamrat Balcha

**Affiliations:** ^1^Department of Medical Laboratory Services, Wolaita Sodo University Comprehensive Specialized Hospital, Wolaita, P.O. Box 138, Ethiopia; ^2^School of Public Health, College of Health Sciences and Medicine, Wolaita Sodo University, Wolaita, P.O. Box 138, Ethiopia; ^3^School of Medical Laboratory, College of Health Sciences and Medicine, Wolaita Sodo University, Wolaita, P.O. Box 138, Ethiopia; ^4^School of Pharmacy, College of Health Sciences and Medicine, Wolaita Sodo University, Wolaita, P.O. Box 138, Ethiopia

## Abstract

**Background:**

Antibiotic resistance is a serious threat to the human population everywhere. However, less attention is given to its concern in sub-Saharan Africa including Ethiopia. There is an information gap concerning antibiotic resistance and its pattern in Wolaita Sodo University Teaching Referral Hospital. This study is aimed at investigating the prevalence of antimicrobial resistance in the study area.

**Methods:**

Five-year retrospective data of cultures and records of 581 patients were utilized to analyze the pattern of antibiotic resistance. The statistical software including SPSS version 25 and Microsoft excel 2013 were used. Laboratory records with incompletely registered age, sex, culture isolation, or drug susceptibility test data were excluded.

**Results:**

Out of the total of 581 samples extracted from the microbiology laboratory, 237 (40.8%) samples were culture positive for bacteria. From positive culture growth, 165 (69.6%) were gram-positive bacteria whereas 72 (30.4%) were gram negative. *Staphylococcus aureus* was the most prevalent isolate among gram-positive isolates as *Escherichia coli* was for gram-negative isolates. Overall antibiotic resistance of gram-positive isolates was 57.2% whereas that of gram-negative bacteria was 58.8%.

**Conclusion:**

*S. aureus* and *E. coli* were found to be the most prevalent pathogenic isolates among gram-positive and gram-negative bacteria, respectively. Most of the isolated pathogens showed high resistance towards the commonly prescribed antibiotic agents. The overall antibiotic resistance in this study was 57.7%, and the overall MDR prevalence was 72.2%.

## 1. Introduction

Antimicrobial resistance (AMR) is one of the major crises of public health and is among the most serious intimidations of the world. This is because it despairs the opportunity of treating morbidities caused by parasites, viruses, fungi, and bacteria [1]. Without any discrimination with respect to economy, age, gender, and/or race [2, 3], it is an eventual ability of these pathogens to resist to the prescribed medicines, the antimicrobials [1, 4, 5]. Many mechanisms can contribute to the resistance: acquisition of resistant genes or mutation in genes that encode for proteins involved, enzymatic hydrolysis, changes in cell membrane response, and/or impermeability [5–7]. Although it may happen naturally, misuse of antibiotics in humans and animals is the leading cause of AMR [4]. The AMR makes the antimicrobials ineffective against the microbial diseases [4, 5]. Multidrug-resistant (MDR) bacteria are bacteria that are nonsusceptible to one or more antibiotic agents in three or more antimicrobial categories whereas “extensive” or “extremely” drug-resistant (XDR) bacteria are those that are nonsusceptible to one or more antibiotic agents in all but two or less antimicrobial classes. When bacteria are nonsusceptible to all antimicrobial agents listed, the bacteria are considered as “pan drug-resistant” (PDR) [8]. The over consumption of antibiotics in general medicine, veterinary, or agriculture has led to a spike in drug-resistant microorganisms [9]. MDR and XDR bacteria have become a major public health threat, and their prevalence in hospital settings is alarmingly increasing [10]. Multiantibiotic-resistant bacteria acquire resistance by mutation and gene transfer via conjunctions, transformation, or transduction [5]. The spread of MDR bacteria from one person to another most often occurs when someone who is colonized with a resistant organism but not ill transmits it to another who then also becomes colonized [10]. The out of pocket money for treating resistant infections is significantly higher than that for nonresistant infections because of longer duration of illness, additional tests, and the need for more expensive medicines [2]. The catastrophe impedes or interferes with the ability to treat infections. It also exerts extremely costly implications cascading to global health, food sustainability, security, environmental wellbeing, product development costs, market failure, and socioeconomic development [11, 12]. Most of its impacts fall on low- and middle-income countries owing to lack of infrastructure and human and financial resources to adequately prevent the drug resistance [12]. The other reasons are high prevalence of infectious diseases, shortage of trained health professionals, irrational use of drugs, and limited microbiological laboratory establishments [2, 3]. In addition, the consequences of AMR are aggravated in situations such as civil unrest, violence, famine, and natural disasters as well as in settings with poor health care services because these situations impair the political momentum of combating AMR [11, 13]. According to WHO report of 2014, in five out of six WHO regions, *Escherichia coli* resistance to third generation cephalosporins and fluoroquinolones and that of *Staphylococcus aureus* to methicillin are higher than 50%. The report claims 45% of deaths in both Africa and South-East Asia were because of multiantibiotic-resistant bacteria. It further reveals that third generation cephalosporin-resistant *K. pneumonia* was cause of high mortality in Africa (77%), Eastern Mediterranean region (50%), South East Asia (81%), and Western Pacific region (72%) [3]. Literature reveals that the rate of AMR varies from region to region and hospital to hospital [14]. Wolaita Sodo University Comprehensive Specialized Hospital is a government hospital that provides general outpatient and inpatient services including medical, surgical, pediatric, psychiatric, ophthalmic, gynecological, and obstetric emergency cares. Annual patient volume is around 200,000. There is no evidence of published work in the literature regarding the burden of antibiotic resistance at this hospital. The current study is aimed at retrospectively investigating the antibiotic resistance pattern of bacteria isolated from different specimens to the commonly prescribed antibiotics at the hospital.

## 2. Materials and Methods

### 2.1. Materials

MacConkey agar, blood agar, urea agar, and chocolate agar manufactured by Sisco Research Laboratories Ltd (India); trypticase Soy broth, Thayer Martin agar, and oxidase manufactured by Oxoid Ltd (UK); lysine iron agar and mannitol salt agar manufactured by Biomark Laboratories Ltd (India); Simmons Citrate Agar and indole manufactured by Himedia Laboratories Ltd (India); catalase (hydrogen peroxide manufactured by Wasse Pharma, Ethiopia); bile esculin agar manufactured by Merck® (Germany); and coagulase manufactured by NVI (Debrezeit, Ethiopia) were the materials used for different purposes and stages of the present study. All the antibiotic discs used including bacitracin (BAC 10 *μ*g), optochin (OPT 5 *μ*g), novobiocin (NOV 30 *μ*g), ampicillin (AM 10 *μ*g), gentamicin (GM 10 *μ*g), cloxacillin (CXC 5 *μ*g), ciprofloxacin (CIP 5 *μ*g), ceftriaxone (CRO 30 *μ*g), nalidixic acid (NA 30 *μ*g), ceftazidime (CAZ 30 *μ*g), cephalexin (CN 30 *μ*g), amoxicillin/clavulanic acid (AMC 20/10 *μ*g), trimethoprim/sulfamethoxazole (SXT 1.25/23.75 *μ*g), chloramphenicol (C 30 *μ*g), tetracycline (TE 30 *μ*g), clindamycin (CM 2 *μ*g), erythromycin (E 15 *μ*g), and vancomycin (VA 30 *μ*g) were manufactured by Abtek Ltd (UK).

### 2.2. Methods

#### 2.2.1. Study Setting and Design

The study was conducted at Wolaita Sodo University Comprehensive Specialized Hospital (WSUTRH) located 329 km to the south of Addis Ababa, capital of Ethiopia. The institution-based five-year (2016-2020) retrospective design was used. Data were collected from June 20/2019 to July 20/2019 and August 1-28 2021. The source population was comprised of all patients who had attended the hospital during 2016-2020, and the study population was all the patients for whom culture had been required and performed during the study period.

#### 2.2.2. Inclusion and Exclusion Criteria

All the encounters' results with complete data registered in the logbook during the study period were included, and all the encounters' results with incomplete data were excluded.

#### 2.2.3. Data Collection

Demographic data of patients pro and files of the bacteria isolated including susceptibility status were retrieved from the microbiology laboratory register books using a standard data collection form. The types of samples which had been collected include urine, stool, body fluid, blood, and pus. These samples had been inoculated accordingly in the culture media and incubated at 37°C based on standard operating procedures, and the growth had been observed after 16-24 hrs.

#### 2.2.4. Microbiological Isolation and Identification

In case blood stream infection was suspected, blood culture media trypticase Soy broth was prepared and blood sample was inoculated aseptically. The samples with hemolysis, gas, and turbidity were subcultured on appropriate media for further isolation. MacConkey and blood agar media were utilized to isolate nonfastidious bacteria whereas chocolate agar media were applied to culture fastidious bacteria. For the samples collected from genital area, Thayer Martin agar media were used. In case growth was observed, a colony from culture was selected and gram stained. Biochemical tests were selected based on their gram reaction. Gram-negative bacteria were further identified using oxidase, citrate, urease, lysine iron agar, mannitol, and indole tests. Gram-positive bacteria were identified using catalase, coagulase, bacitracin, optochin, bile solubility, and novobiocin. By using gram staining, identification of some bacteria was performed up to species level for some and genus level for the others.

#### 2.2.5. Antibiotic Resistance Testing

Antibiotic resistance test was conducted, and interpretation was performed based on the standard Kirby-Bauer disc diffusion method of Clinical Laboratories Standard Institute (CLSI) 2014–2017. The test results were reported based on whether the isolates were resistant towards recommended doses of the antibiotics for the site of infection. The “resistant” category implied that isolates were not inhibited by the usually achievable concentrations of the agent with normal dosage schedules. Another implication of “resistant” category was demonstration of minimum inhibitory concentration (MIC) or zone diameters that fall in the range where specific microbial resistant mechanisms are likely, and clinical efficacy of the agent against the isolate was not reliably shown in treatment studies.

#### 2.2.6. Quality Assurance

Before testing all the inoculated samples, a standard bacteriological procedure was followed to maintain correct laboratory test results. American Type Culture Collection (ATCC) standard reference strains of *Escherichia coli* (ATCC-25922), *Staphylococcus aureus* (ATCC 25923), and *Pseudomonas aeruginosa (*ATCC-25853) were used to control the quality of the culture and drug susceptibility testing. All data were checked for consistency and completeness.

#### 2.2.7. Ethical Considerations

The ethical clearance was obtained from the ethical review committee of Wolaita Sodo University, College of Health Science and Medicine. Formal consent was also obtained from Wolaita Sodo University Referral Hospital. For privacy reason, all data was kept confidential. Anonymity of records was maintained by using registration number and unique code numbers used by service providers at Wolaita Sodo University Comprehensive Specialized Hospital.

#### 2.2.8. Data Processing and Analysis

The data were cleaned and analyzed by using SPSS Version 25 and Microsoft excel 2013 software. The results were summarized using descriptive presentations like frequency and percent distributions when appropriate [15].

## 3. Results and Discussions

### 3.1. Sociodemographic Characteristics of the Subjects

**Figure 1:**
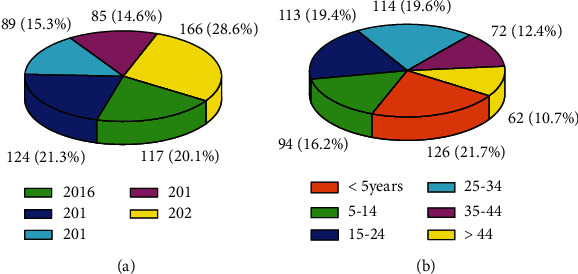
(a) Year of testing and (b) age group of patients involved in antibiotic resistance study at WSUCSH, 2016-2020.

**Figure 2:**
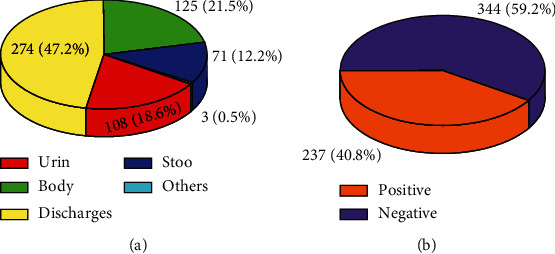
(a) Types and the (b) overall growth rate in the specimens inoculated for antibiotic resistance study at WSUCSH, 2016-2020.

Out of total of 581 encounters, males were 304 (52.3%) and 277 (47.7%) were females. From the total of 581 patients, 166 (28.6%), 124 (21.3%), and 117 (20.1%) visited the microbiological laboratory in the year 2020, 2017, and 2016, respectively (Figure 1(a)). The majority of the age groups, 126 (21.7%), were below five years, and the age group least in number was 45 years and older, 62 (10.7%) (Figure 1(b)). Similarly, the majority, 47.2%, of specimens used for culturing were body discharges and pus (Figure 2). This comparative distribution was in agreement with a report from Yemen [16].

### 3.2. Growth of Isolates

**Figure 3:**
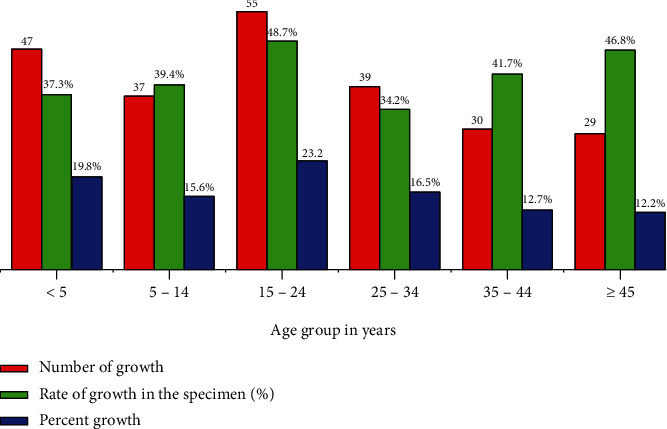
Distribution of growth of bacterial isolates among different age groups from which the specimens were collected.

Out of the total specimens inoculated, 237 (40.8%) showed positive bacterial growth whereas 344 (59.2%) did not show any growth (Figure 2(b)). This was significantly lower than the rate of growth reported from Gondar, 70.2%. The reason for the lower rate might be inefficient screening of the specimens [17]. Distribution of growth status of bacteria in the different age groups is shown in Figure 3. The age group with highest number of growth, 55, was 15-24 years which contributed 23.2% of the total growth. The rate of bacterial growth for this group was 48.7% which indicates the highest efficiency of screening of encounters for culture. In contrast, the age group with least number of growth, 29, was ≥44 years which contributed 12.2% of the total growth. The rate of bacterial growth in the specimens from the oldest group was 46.8% which could be taken as the second highest efficiency of screening of encounters for culture. The least efficiency of screening of the encounters for the culture, 34.2%, was observed in the age group 25–34 years.

Concerning the sources of specimens used for bacterial isolation, 274 (47.2%) were discharges from vagina, urethra, and wound; 71 (12.2%) were from stool; 108 (18.6%) were from urine, 125 (21.5%) were from body fluids, and 3 (0.5%) were from others (Figure 2(b)). The top three bacteria isolated from the positive growths in this study were *S. aureus* 132 (55.7%), *E. coli* 30 (5.2%), and *P. aurogenous* 27 (4.6%). That *S. aureus* and *E. coli* were successively the most prevalent isolates was consistent with studies reported elsewhere in Ethiopia [2, 18]. Among those 237 positive growths, based on their gram reaction, 165 (69.6%) were gram positive and 72 (30.4%) were gram negative. This was in contrast to the studies conducted elsewhere where the rate of growth was higher for gram-negative isolates than the gram-positive ones [19, 20]. The reason might be the higher proportion specimens pertaining to body discharges and pus which are the most common sites of S. aureus manifestations [21] and/or variation of bacterial etiology across geographical conditions. Gram-positive bacteria identified were *S. aureus*, *S. pyogenes*, *S. pneumonia*, and *S. saprophytes* whereas gram-negative bacteria isolated include *E. coli*, *P*. *aurogenous*, *Proteus species*, *Salmonella species*, *Shigella species*, and *Neisseria species* (Table 1). Differences in species prevalence rates might happen by virtue of differences in places of specimen collection or their distribution in the various environments.

### 3.3. The Antibiotic Resistance of Bacterial Isolates

The resistance of gram-positive bacteria to the tested antibiotics is shown in Table 2. Its values ranged from 55.7% of S. aureus to 64.6% of S. saprophytes, and its average value was 57.2% whereas the overall resistance in this study was 57.7%. This value was lower than that conducted in Debre Markos Referral Hospital, Ethiopia, 84.6% for gram-positive bacteria [22–24] which might be due to some updates in microbiological set-ups and better awareness of prescribers [25]. The most prevalent isolate among all the bacterial growths in this study was *S. aureus*, 132 (55.7%), which is consistent with the findings from the study conducted in Gabon, Central Africa [2]. It was resistant to many of the antibiotics tested including ampicillin, tetracycline, vancomycin, and chloramphenicol. In this regards, the findings agree to the findings reported from elsewhere [18, 20, 26]. Its resistance to the tested antibiotics ranged from 15 to 86% with the overall rate of 55.7% which is lower than the report from Greek where it was 88% [27]. On the other hand, it was higher than the report of resistance, 40%, observed in patients with suspected peritonitis in Southern Ethiopia [28] and the 3 years retrospective study report from the same region in which the resistance of *S. aureus* was 42.02% [23] to the respective antibiotics. Its resistance was highest to ampicillin (86.0%), 74.2% to vancomycin, 66.1% to ceftriaxone, 55.7% to cephalexin, 68.8% to cloxacillin, 73.4% to chloramphenicol, and 48.3% to erythromycin. The resistance of gram-positive bacterial isolates was highest to ampicillin and tetracycline which is in line with studies conducted elsewhere in Ethiopia and Nigeria [20, 29]. The resistance towards ampicillin in this study was extremely higher than that reported for Enterococci elsewhere in Ethiopia [30]. The overall resistance towards ampicillin was lower than the reports from elsewhere [31, 32] but significantly higher than the other reports [16]. The reason for high resistance towards ampicillin and also others might be due to overuse of these antibiotics [33]. The overall resistance of *S. pyogenes* and *S. pneumonia* were found to be 58.8% and 62.8%, respectively. The highest resistance of *S. pyogenes* was observed for ceftriaxone. The probable reason for this finding might be extreme and nonprioritized overuse of ceftriaxone. The lowest resistance of *S. pyogenes* was observed for ciprofloxacin and erythromycin. The overall resistance of tested gram-positive bacteria was 82.2% to ampicillin, 75.6% to tetracycline, 73.7% to chloramphenicol, and 73.5% to gentamicin. The resistance of erythromycin was similar to the findings from elsewhere [34]. The overall resistance of the bacterial isolates towards vancomycin was 72.2% which was in line with the report that vancomycin resistance is rising in Ethiopia [30]. The reason for vancomycin resistance had been scrutinized to be availability of encoding system for the synthesis of low affinity precursors and elimination of high affinity precursors [35]. It notifies a great threat to the public health because vancomycin is the medicine reserved for seriously ill patients or for penicillin, cephalosporin, and other antibiotic-resistant infections [36].

The antibiotic resistance of gram-negative bacteria isolated towards commonly prescribed antibiotics is shown in Table 3. The overall resistance of gram-negative bacteria in this study (58.8%) was lower than that reported from southern Ethiopia University Teaching Hospitals, 84.0% [23]. The least resistance of the gram-negative bacterial isolates, 55.6%, was observed for *Salmonella* which was comparable to that of *E. coli*, 55.8%. In contrast, the highest resistance was that of *E. proteus*, 63.2%, which was slightly more resistant than *N. meningitis* (60.0%). The overall resistance of *E. coli* ranged from 19% of nalidixic acid to that of tetracycline, 89%. Its overall resistance was 55.8%, and in the meta-analysis conducted in Ethiopia, it was 45.38% (33.5%-57.7%), the highest resistance in Addis Ababa and the least in the Tigray Region [37]. The specific rate of resistance of *E. coli* to cotrimoxazole was 80.0%, ceftriaxone was 70%, chloramphenicol was 50.0%, and gentamycin was 61.1%. Its resistance was relatively lowest, 18.8%, to nalidixic acid. The findings of this study could be taken as comparable to the previous studies conducted in Ethiopia and Egypt [23, 38, 39]. The overall resistance (56.7%) of gram-negative isolates to ceftriaxone was also lower than that reported from the study in Rewand Referral Hospital where out of 241 gram-negative isolates tested for ceftriaxone, 75.9% had been resistant [40]. The resistance of *Salmonella* species towards the overall antibiotics tested was 55.6% which was comparable with 54.55% which was the resistance of the same species reported from elsewhere in Ethiopia [31]. In contrast, it was lower than the report from Bangladesh, 62.94% [41]. The reason for this difference might be better prescribing and using practice of antibiotics, geographical variation, or the effect of sample size. The overall resistances of *P. aeruginosa and Shigella* species were revealed to be 63.9% and 61.1%, respectively. *P. aeruginosa* isolates were resistant to tetracycline (91.7%), ampicillin (66.7), and cotrimoxazole (60.0%). Comparable findings were also reported elsewhere from Ethiopia [28, 41, 42]. In addition, that it had lower resistance to ciprofloxacin was in line with the literature evidences [26, 43]. However, according to the present study, *P. aeruginosa* was resistant to gentamycin and ceftriaxone too and the prevalence of its overall resistance is rising.

Overall, most of the isolates including both gram-positive and gram-negative bacteria were found to be resistant to the majority of the antibiotics. The same findings were reported in the literature elsewhere from Ethiopia [44, 45]. The average resistance of all the tested bacteria against ampicillin was 80.8% which was significantly higher than that reported elsewhere [16]. The reason might be overuse of the medicine. Upon analysis of the individual bacteria for resistance, *S. saphropytic*, *S. pneumonia*, *P. aurogenous*, *Salmonella*, *Shigella*, and *E. proteus* relatively had a larger overall proportion of resistance to the antibiotics. This finding is in agreement with a study reported from Hawassa where antibiotic resistance of gram-negative bacteria was higher than that of the gram-positive bacteria [23]. Ampicillin, tetracycline, chloramphenicol, and gentamicin were the first five successive antibiotics to which the highest gram-positive bacteria resistance was observed. Similarly, tetracycline, ampicillin, cloxacillin, and cotrimoxazole were the antibiotics to which the highest record of gram-negative bacteria resistance was observed successively in decreasing order. The resistance rate to some antibiotics in this study was much higher than a recent study from other parts of Ethiopia. For instance, the resistance to penicillins and tetracyclines was in the range of 35-47% and 38-52% in gram-positive bacteria, respectively. Also, the resistance rate of gram-positive bacteria to ceftriaxone was very lower (20%), compared to the present study (69%). However, interestingly, the resistance rate to fluoroquinolones was lowest in both bacteria groups, and the resistance rate of gram-negative bacteria to penicillin's and ceftriaxone was comparable in both settings [46]. On the other hand, the resistance reported from the present study is higher than that reported by the studies conducted in the African countries which revealed antibiotic resistance of 34.6% in Benin, 31.9% in Congo, 14.3% in Togo, and 16.3%in Madagascar [47]. The overall resistance in the present study (57.7%) was lower than that reported from Central Ethiopia (72.2%) and also Debre Markos Referral Hospital, Ethiopia, 72.2% [22, 24, 48]. This could be due to some updates in diagnostic set-ups and better awareness of prescribers on rational prescribing [25]. It might also be because of differences in the geographical area, type of organisms, and the methods used.

### 3.4. Multidrug Resistance (MDR)

The overall prevalence of MDR as per the present study was 72.2% which was significantly higher than that of Tigray (51.1%) and Amhara (68.6%). It was slightly higher than that of pooled MDR of the overall Ethiopia (70.5%) and the report of Oromia Region (70.1%). Still it was comparable to that of Addis Ababa (72.4%) but slightly lower than that of Harari (74.6%) and significantly lower than that of Sidama (81.7%). The differences in the MDR prevalence might be due to the differences in geography, client types, the types of bacteria, the methods used, and variations in the level of implementation of infection prevention protocols [49]. The findings showed the MDR prevalence in the present study was higher than the report from elsewhere in Ethiopia, 47.8% [23]. The mean prevalence of MDR in gram-positive and gram-negative bacteria in the present study was 73.9% and 68.1, respectively. This shows the prevalence of MDR among the gram-positive bacterial isolates in this study was higher than the report from Bangladesh where it was 68.8% [50]. As indicated in Table 4, the gram-positive bacteria including *S. saprophyte*, *S. pneumonia*, and *S. pyogenes* experienced the highest MDR levels (100%) successively followed by *P. aeruginosa* (88.9%) and *S. aureus* (67.4%). Likewise, the prevalence of MDR in gram-negative bacteria was higher than the report of pooled MDR from elsewhere, 27% [51]. The MDR of each of *E. coli* and *Salmonellae* was 50% which was the lowest finding in the present study. This shows that the MDR of *E. coli* was lower than the report from Nigeria where its value was 88%. In addition, the *Proteus* species showed higher MDR in this study than that of the same study in Nigeria, 60% [52]. The reasons for the differences might be the differences in the types of bacteria tested, geographical conditions, profiles of patients, and practice of rational use of medicines. In general, the implication was that, in the study area, the effectiveness of treatment of bacterial infections with the commonly prescribed antibiotics could have been significantly affected by nonsusceptibility. In this regards, the antibiotic resistance has already become a great challenge in the study area requires further investigations and intervention.

## 4. Limitation of the Study

Since it is a retrospective study, detailed sociodemographic information and clinical status of patients were not incorporated. The number of drugs tested on some pathogens was small which could probably affect the representativeness. The total number of encounters was also less than optimum that it could probably have affected the findings. That the correlation of Kirby-Bauer zone of inhibition data with the minimum inhibitory concentration experimental data might also be taken as a limitation.

## 5. Conclusions and Recommendations

According to the present study, the most frequent bacterial isolates were *S. aureus*, *E. coli*, *P. aurogenous*, *S. saprophytes*, *and S. pyogenes*, in decreasing order. The commonly prescribed antibiotics would have questionable effectiveness for claimed therapeutic indications in the study area. The overall antibiotic resistance in this study was 57.7%. Its prevalence in gram-positive bacteria ranged from 55.7% of *S. aureus* to 64.6% of *S. saprophytes*, and its average value was 57.2%. The overall prevalence of MDR as per the present study was 72.2%. The MDR prevalence in the study area was higher than many reports from elsewhere in Ethiopia. The mean prevalence of MDR in gram-positive and gram-negative bacteria in the present study was 73.9% and 68.1, respectively. In this regards, the antibiotic resistance has been posing and will continue to pretense even more challenges to the public health in the study area unless proportional interventions are considered. In order to reveal impact and routes of antibiotic resistance, further studies including resistant gene identification should be conducted. The reasonable recommendations would comprise enthusiastic implementation of infection control protocols and antibiotic stewardship augmented with interdisciplinary collaboration of health care providers.

## Figures and Tables

**Table 1:** Types and distribution of isolated bacteria at WSUTRH, 2016-2018.

Bacteria		Frequency	Percentage (%)
Gram-positive bacteria	*S. aureus*	132	55.7
	*S. pyogenes*	12	5.1
	*S. pneumonia*	7	3.0
	*S. saprophytes*	14	5.9
	Subtotal	165	69.6

Gram-negative bacteria	*P. aurogenous*	27	11.4
	*E. coli*	30	12.7
	*Proteus spp.*	5	2.1
	*Shigella*	5	2.1
	*Neisseria spp.*	3	1.3
	*Salmonella*	2	0.8
	Subtotal	72	30.4
	Total	237	100%

**Table 2:** The antibiotic resistance of gram-positive bacterial isolates.

Antibiotic tested	*S. aureus*		*S. pyogenes*		*S. saphropytic*		*S. pneumonia*		Total (%)
	R/T	%R	R/T	%R	R/T	%R	R/T	%R	
AM	49/57	86.0	5/8	62.5	4/5	80.0	2/3	67	60 (82.2)
C	58/79	73.4	4/7	57.1	7/9	77.8	4/4	100	73 (73.7)
GM	52/78	66.7	8/10	80.0	8/9	88.9	4/5	80	72 (73.5)
CXC	55/80	68.8	8/9	88.9	8/11	72.7	3/4	75	74 (71.2)
CRO	72/109	66.1	10/11	90.9	11/14	78.6	4/6	67	97 (69.3)
CIP	18/121	14.9	1/11	9.1	5/14	36	0/7	0	24 (15.7)
CL	31/105	29.5	4/9	44.4	4/13	31	3/6	50	42 (31.6)
SXT	19/31	61.3	4/6	66.7	6/7	86	2/3	67	31 (66.0)
TE	22/29	75.9	3/5	60.0	4/5	80	2/2	100	31 (75.6)
E	28/58	48.3	1/7	14.3	6/8	75	1/2	50	36 (48.0)
CN	34/61	55.7	5/6	83.3	4/8	50	1/3	33	44 (56.4)
VA	49/66	74.2	4/8	50.0	6/10	60	6/6	100	65 (72.2)
Overall	487/874	55.7	57/97	58.8	73/113	64.6	32/51	62.8	649 (57.2)

AM: ampicillin; C: chloramphenicol; GM: gentamycin; CXC: cloxacillin; CTO: ceftriaxone; CIP: ciprofloxacin; CL: clindamycin; SXT: trimethoprim/sulfamethoxazole; TE: tetracycline; E: erythromycin; CN: cefalexin; VA: vancomycin; R: resistant; T: number of tests conducted; %R: percent *resistance.*

**Table 3:** The antibiotic resistance of gram-negative bacterial isolates.

Antibiotic tested	*E. coli*		*P. aurogenous*		*Salmonella*		*Shigella*		*Proteus*		*N. meningitis*		Total (%)
	R/T	%R	R/T	%R	R/T	%R	R/T	%R	R/T	%R	R/T	%R	
AM	9/11	81.8	8/12	66.7	1/1	100.0	2/2	100.0	3/4	75.0	1/1	100.0	24 (77.4)
C	11/22	50.0	8/14	57.1	1/1	100.0	1/2	50.0	3/5	60.0	0/2	0	22 (47.8)
GM	11/18	61.1	11/18	61.1	1/2	50.0	1/2	50.0	2/5	40.0	1/3	33.3	27 (56.3)
CXC	8/14	57.1	16/19	84.2	-	-	1/1	100.0	2/2	100.0	-	-	27 (75.0)
CRO	16/23	69.6	22/26	84.6	-	-	1/5	20.0	3/4	75.0	2/2	100.3	34 (56.7)
NA	3/16	18.8	8/20	40.0	0/1	0	1/2	50.0	2/3	66.7	-	-	14 (33.3)
CPR	8/25	32.0	9/26	34.6	-	-	2/5	40.0	2/5	40.0	2/3	66.7	23 (36.0)
CM	13/19	68.4	14/22	63.6	1/1	100.0	3/3	100.0	2/3	66.7	0/1	0	33 (67.3)
SXT	12/15	80.0	9/15	60.0	-	-	2/3	66.7	1/1	100.0	1/1	100.0	25 (71.4)
TET	8/9	88.9	11/12	91.7	1/1	100.0	3/3	100.0	3/3	100.0	1/1	100.0	27 (93.1)
ERY	5/13	38.5	8/16	50.0	0/1	0	0/1	0	1/2	50.0	1/1	100.0	15 (44.1)
CN	7/14	50.0	4/10	40.0	0/1	0	1/2	50.0	0/1	0	-	-	12 (42.9)
Overall	111/199	55.8	128/210	61.0	5/9	55.6	18/31	58.1	24/38	63.2	9/15	60.0	295 (58.8)

AM: ampicillin; C: chloramphenicol; GM: gentamycin; CXC: cloxacillin; CRO: ceftriaxone; NA: nalidixic acid; CIP: ciprofloxacin; CM: clindamycin; SXT: trimethoprim/sulfamethoxazole; TE: tetracycline; E: erythromycin; CN: cefalexin; R: resistant; T: number of tests conducted; %R; percent resistance.

**Table 4:** Multiple antibiotic resistance patterns of bacterial isolates in WSUCSH, 2016–2020.

Bacteria	*R*_0_		*R*_1_		*R*_2_		*R*_3_		*R*_4_		*R*_5_ ≥		Total		MDR	
	*N*	%	*N*	%	*N*	%	*N*	%	*N*	%	*N*	%	*N*	%	*N*	%
*S. aureus*	6	4.5	10	7.6	27	20.5	24	18.2	21	15.9	44	33.3	132	100	89	67.4
*S. saprophyte*	0	0.0	0	0.0	0	0.0	4	28.6	2	14.3	8	57.1	14	100	14	100.0
*S. pneumonia*	0	0.0	0	0.0	0	0.0	3	42.9	2	28,6	2	28.6	7	100	7	100.0
*S. pyogenes*	0	0.0	0	0.0	0	0.0	5	41.7	2	16.7	5	41.7	12	100	12	100.0
Subtotal	6	4.5	10	7.5	27	16.4	36	21.8	27	16.4	59	35.8	165	100	122	73.9
*P. aeruginosa*	0	0.0	1	3.7	2	7.4	8	29.6	9	33.3	7	25.9	27	100	24	88.9
*E. coli*	2	6.7	3	10	10	33.3	3	10.0	3	10.0	9	30.0	30	100	15	50.0
*Proteus spp.*	0	0.0	0	0.0	2	40	0	0	0	0.0	3	60.0	5	100	3	60.0
*Shigella*	0	0.0	1	20.0	0	0.0	1	20.0	2	40.0	1	20.0	5	100	4	80.0
*Salmonella*	0	0.0	0	0.0	1	50.0	1	50.0	0	0.0	0	0.0	2	100	1	50.0
*N. meningitis*	0	0.0	0	0.0	1	33.3	1	33.3	0	0.0	1	33.3	3	100	2	66.7
Subtotal	2	2.8	5	6.9	16	22.2	14	19.4	14	19.4	21	29.2	72	100	49	68.1
Total	8	3.4	15	6.3	43	18.1	50	21.1	41	17.3	80	33.8	237	100	171	72.2

## Data Availability

The authors confirm that all the data associated with this paper are available upon request.

## Abbreviations

AMR: Antimicrobial resistance
ATCC: American Type Culture Collection
MDR: Multidrug resistance
MIC: Minimum inhibitory concentration
SNNPR: South Nations, Nationalities, and Peoples Region
spp.: Species
WHO: World Health Organizations
WSUTRH: Wolaita Sodo University Teaching Referral Hospital.
